# Characterization of viable but nonculturable state of *Campylobacter concisus*

**DOI:** 10.1098/rsos.240088

**Published:** 2024-05-29

**Authors:** Syeda Umme Habiba Wahid, Bronwyn E. Campbell, Robert J. Moore, Taghrid Istivan

**Affiliations:** ^1^ School of Science, RMIT University, Bundoora, Victoria, Australia

**Keywords:** *Campylobacter concisus*, viable but nonculturable (VBNC), resuscitation, PMAxx-qPCR, genomospecies

## Abstract

*Campylobacter concisus* is an opportunistic bacterial pathogen linked with a range of human diseases. The objective of this study was to investigate the viable but nonculturable (VBNC) state of the bacterium. To induce the VBNC state, *C. concisus* cells were maintained in sterilized phosphate-buffered saline at 4°C for three weeks. The VBNC cells were monitored using quantitative analysis by propidium monoazide (PMAxx) coupled with quantitative real-time PCR (PMAxx-qPCR), targeting the *DNA gyrase* subunit B gene. The results demonstrated that *C. concisus* ATCC 51562 entered the VBNC state in 15 days, while ATCC 51561 entered the VBNC state in 9 days. The viable cell counts, assessed by PMAxx-qPCR, consistently remained close to the initial level of 10^7^ CFU ml^−1^, indicating a substantial portion of the cell population had entered the VBNC state. Notably, morphological analysis revealed that the VBNC cells became coccoid and significantly smaller. The cells could be resuscitated through a temperature increase in the presence of a highly nutritious growth medium. In conclusion, under environmental stress, most *C. concisus* cells converted to the VBNC state. The VBNC state of *C. concisus* may be important for its environmental survival and spread, and the presence of VBNC forms should be considered in environmental and clinical monitoring.

## 1. Introduction

Microorganisms continually face dynamic environmental conditions, necessitating them to employ diverse survival strategies through adaptive adjustments in their physiology. This adaptation allows them to optimize resource utilization while preserving their structural and genetic integrity, ultimately enhancing their resilience and tolerance to adverse conditions. One crucial survival strategy observed in some bacteria is the viable but nonculturable (VBNC) state, first reported in 1982 by Xu *et al.* [1]. Bacteria can enter a VBNC state when encountering harsh environmental conditions such as extremes of temperature, osmolarity and pH, and the presence of toxic chemicals [2–4]. Currently, over 100 bacterial species spanning 50 different genera have been reported to enter the VBNC state [3,5–8]. It is known that bacteria in the VBNC state have reduced metabolic activity and cannot be detected by traditional culture-based methods, which could lead to a severe underestimation of potential risks to public health [9].

In recent years, numerous studies have highlighted the remarkable capability of microorganisms to recover from a dormant state and regain full metabolic activity and culturability under specific environmental conditions [3,10]. This phenomenon has garnered significant attention and has been extensively documented. Interestingly, previous studies have reported that some human pathogens could retain pathogenicity in the VBNC form [2,11,12]. The underlying mechanisms of resuscitation remain unclear. Nevertheless, certain factors promoting the restoration of culturability have been identified, including upshifting of temperature in the presence of nutrients, pyruvate, glutamate, gas mixture, vitamins and amino acids [13]. Also, pyruvate, acting as an antioxidant, has been recognized as a key promoter of resuscitation by scavenging hydrogen peroxide and hydroxyl radicals and preventing lipid peroxidation [14]. Another study reported that *Campylobacter jejuni* VBNC cells were recovered and grown using a combination of ferrous sulfate, sodium metabisulfite and sodium pyruvate in the culture media [15]. Yet, little information is available regarding the VBNC state and resuscitation of VBNC cells in *Campylobacter concisus.*

The Gram-negative bacterium *C. concisus* was first identified and isolated by Tanner *et al.* [16] from the oral cavity of patients with gingival–periodontal disease. This opportunistic pathogen commonly lives in the human oral cavity [17]. *C. concisus* strains can also be isolated from different sites within the gastrointestinal (GI) tract of humans [18]. Furthermore, it is associated with many diseases, including prolonged diarrhoea, gingival inflammation and Barrett’s oesophagus [17–19]. Based on comprehensive genomic analysis, *C. concisus* has been grouped into two genomospecies (GS), GS1 and GS2. They share similar genetic characteristics but display variations in their virulence, pathogenic potential and adaptation capabilities in the human GI tract [20–23].

Culture-dependent or molecular methods are currently used to detect *C. concisus*. Several studies have used a membrane filter-based culture method to isolate this bacterium [24,25]. The isolation of *C. concisus* through traditional culture-based methods often leads to an underestimation of its abundance [26]. To overcome this limitation, various cultivation-independent techniques have been employed for its detection. These techniques encompass a range of approaches, such as PCR, enzyme-linked immunosorbent assay and loop-mediated isothermal amplification [25,27]. However, the DNA-based molecular methods (PCR and quantitative PCR (qPCR)) overestimate prevalence owing to false-positive results by amplifying DNA from dead cells [28]. These methods cannot differentiate between live and dead cells. The quantitative detection of viable bacteria becomes more complicated when cells have entered the VBNC state.

Prior to conducting qPCR assays, pre-treatment of samples with nucleic acid intercalating dyes has been proposed as an effective technique to mitigate false-positive results. Ethidium monoazide bromide, propidium monoazide (PMA) and its improved derivative (PMAxx) are examples of such dyes [28,29]. This pre-treatment facilitates the selective detection of only DNA from viable cells, including the culturable and VBNC microorganisms. The differentiation is based on the ability of PMAxx to penetrate dead or membrane-compromised cells and crosslink covalently to DNA after exposure to intense light. The PMAxx-bound DNA is insoluble, and its amplification by qPCR is inhibited [28]. Thus, it has been successfully integrated with qPCR assays for the detection of viable cells from different bacterial species from a wide range of samples [5,9,28,30–32]. Hence, its use is extended to study the VBNC state of *C. concisus*.

The present study aimed to induce the VBNC state of *C. concisus* under nutrient starvation conditions and resuscitate cells into the actively metabolizing state using modified culture media to investigate their morphological transformation. Within this investigation, an assay was developed that used the PMAxx-qPCR method to assess the presence of *C. concisus* viable cells in both fresh highly viable cultures and in cultures converted to the VBNC state. The results were compared with enumeration obtained by traditional qPCR and plate count methods.

## 2. Material and methods

### 2.1. Bacterial strains and growth conditions

*C. concisus* strains ATCC 51561 and ATCC 51562 were used in this study [33]. Bacterial cells were cultured on modified Columbia agar (CA) plates supplemented with 5% horse blood and 0.4% Na-fumarate under H_2_-enriched microaerobic conditions (5% O_2_, 5% H_2_, 10% CO_2_ and 80% N_2_) [22,34]. Bacterial strains were stocked in tryptone/skim milk medium (1% (w/v) tryptone and 10% (w/v) skimmed milk) at −80°C until assayed.

### 2.2. Preparation of heat-killed cell suspensions

Bacterial cells grown on CA plates were harvested and resuspended into sterile phosphate-buffered saline (PBS, pH 7.4) to a concentration of 10^7^ CFU ml^−1^. One millilitre of cell suspension was subjected to heat treatment at 90°C for 5 min to kill the cells. The effect of the heat killing was confirmed by spreading 100 μl of the suspension on CA plates and incubating them at 37°C for 72 h under microaerobic conditions. The absence of colony formation confirmed the effectiveness of the heat treatment.

### 2.3. Optimization of propidium monoazide concentration

An improved version of photoactivable DNA binding dye, PMAxx, was acquired from Biotium (USA). PMAxx was added to bacterial suspensions at varying concentrations, and the mixture was incubated in the dark with constant shaking at 150 rpm for 10 min. Subsequently, DNA crosslinking was performed by exposing the tubes horizontally to a 500 W halogen light at a distance of 20 cm for 15 min. The mixture was then centrifuged at 15 000 × *g* for 10 min, and a single wash with sterile distilled deionized water was performed to eliminate any remaining PMAxx before DNA extraction. The optimal concentration of PMAxx required for qPCR experiments was determined using live and heat-inactivated *C. concisus* cells. Separate samples containing 10^7^ CFU ml^−1^ of live or heat-inactivated cells were treated with PMAxx at concentrations of 0, 10, 20, 30, 40 and 50 µM prior to DNA extraction.

### 2.4. Real-time quantitative PCR amplification

Determination of total cell number and viable cells were performed using qPCR and PMAxx-qPCR, respectively. Primers targeting the *gyrB* gene, which encodes the subunit B protein of DNA gyrase, were used for *C. concisus* detection, as previously described [35]. The primer sequences used were as follows: Pcisus5-F (5′-AGCAGCATCTATATCACGTT-3′) and Pcisus6-R (5′-CCCGTTTGATAGGCGATAG-3′). The qPCR reactions were performed using a total volume of 12 µl, comprising 1× SensiFAST SYBR Mix (Bioline), 5 µl of DNA template, 100 nM of each primer and sterile water as required. The qPCR experiments were conducted using a CFX Connect Real-Time System (Bio-Rad). To account for potential contamination, a negative control (ddH_2_O) was included in each qPCR run, and triplicate testing was performed for each sample. The temperature cycle started with 300 s at 95°C (initial polymerase activation) followed by 40 cycles of 15 s at 95°C (denaturation), 15 s at 55°C (annealing) and then 60 s at 72°C for extension. The PCR generated an amplicon of 344 bp.

### 2.5. Construction of standard curve from genomic DNA

To establish a standard curve to be used in determining the efficiency of qPCR, a 10-fold serial dilution of genomic DNA from *C. concisus* ATCC 51562 was prepared in ultrapure water, resulting in final DNA concentrations equivalent to a cell content ranging from 10^0^ to 10^8^ CFU ml^−1^. The efficiency, slope and correlation coefficient of the standard curves were determined by plotting the Cq values against the log_10_ CFU ml^−1^. The amplification efficiency was calculated using the equation *E* = [10^(−1/*s*)^ − 1] × 100%, where *E* represents the efficiency as a percentage and *s* denotes the slope obtained from the standard curve. The limit of detection (LOD) for this assay was defined as the lowest CFU ml^−1^ of *C. concisus* that yielded Cq values below 35. Each run of the standard curve was conducted in triplicate, and the mean standard curve was calculated by averaging the cycle threshold (Cq) values and their respective standard deviations (s.d.) across the triplicate measurements.

### 2.6. Induction of viable but nonculturable state

**Figure 1.**
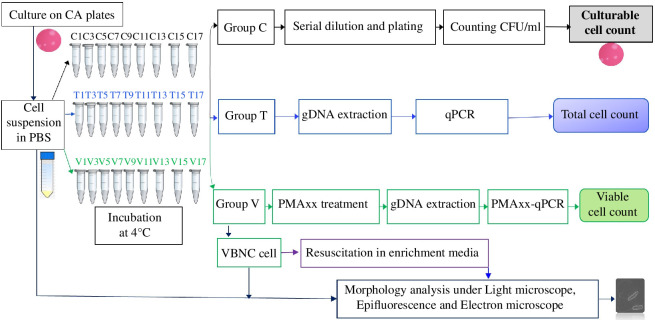
Experimental flow diagram of *C. concisus* cold stress VBNC induction and assay for quantification by plate counting (Group C), qPCR (Group T) and PMAxx-qPCR (Group V). Cell morphology was assessed using light microscopy, epifluorescence and transmission electron microscopy.

The induction of the VBNC state at low temperature under limited nutrient conditions was performed following the protocol used for *C. jejuni* with some modifications [5,36]. *C. concisus* cells were washed and resuspended in PBS to a concentration of 10^7^ CFU ml^−1^. The cell suspensions were divided into three sets (Group C, culturable cells; Group T, total cell count; and Group V, VBNC cell count) and kept at 4°C to induce a VBNC state (figure 1). Entry of cells into the VBNC state was monitored by using three methods: conventional culture method, qPCR and PMAxx-qPCR. On every second day, samples were removed, vortexed briefly and tested.

### 2.7. Determination of culturable, total and viable but nonculturable cell number

The culturable cell number (Group C) was counted every second day for 23 days by a traditional plate counting method. Briefly, a 10-fold serial dilution was performed in 0.85% (w/v) sterile NaCl solution and 0.1 ml aliquots were spread on CA plates and incubated at 37°C for 48 h under microaerophilic conditions. Colonies were then counted to calculate CFU ml^−1^. The second set of samples (Group T) remained untreated and were used for the overall cell quantification by qPCR. The third set of samples (Group V) underwent PMAxx treatment under optimal conditions. The cell pellets from both the second and third groups were washed with 0.85% (w/v) normal saline prior to genomic DNA extraction (figure 1). When the culturable cell concentration was <1 CFU ml^−1^, it was considered that all the viable cells quantified by PMAxx-qPCR were VBNC cells. As validation, 0.1 ml of the putative VBNC cell suspensions was spread on CA plates and incubated under the optimum microaerophilic conditions for 72 h to confirm that no cells could be recovered.

### 2.8. Resuscitation of viable but nonculturable cells

Upon loss of culturability on CA, the samples underwent a recovery process for further analysis. This involved subjecting the samples to temperature upshift and enrichment in Bolton Broth (BB) followed by plating on supplemented agar. This method has been previously used in *C. jejuni* [36]. In brief, to initiate the resuscitation process, a 1 ml aliquot of the cell suspension was combined with 5 ml of sterile BB (Oxoid CM0983) and incubated under microaerobic conditions at 37°C with gentle agitation for 48 h. Following the enrichment period, a 0.1 ml portion of the suspension was plated on supplemented CA agar, which was then incubated at 37°C for 48 h prior to quantification.

Pyruvate is known to be a factor that promotes the resuscitation of VBNC cells [14]. A combination of ferrous sulfate, sodium metabisulfite and sodium pyruvate has been reported to promote *C. jejuni* viability in culture media [37]. Therefore, the supplemented CA was prepared by incorporating *Campylobacter* agar base (Oxoid CM0689) along with *Campylobacter* growth supplement (Oxoid SR0232), comprising ferrous sulfate, sodium metabisulfite and sodium pyruvate.

### 2.9. Cell morphology analysis

Cell morphology was investigated using various microscopic techniques. Initially, cells were stained with the LIVE/DEAD *Bac*light Bacterial Viability Kit (L13152, Invitrogen) and observed under a confocal laser scanning microscope. Additionally, the examination of cell morphology was performed using a transmission electron microscope. To prepare the samples for transmission electron microscopy, bacterial cells were cultivated in Columbia broth. A carbon-coated parlodion film-covered grid was gently placed onto a 20 μl sample drop, allowing the sample to adsorb to the grid for 2 min. Subsequently, the grid was carefully lifted from the drop and transferred onto a 1% phosphotungstic acid solution (pH 7.0) for 1 min. To remove excess stain, the grid was carefully touched against a piece of Whatman filter paper, ensuring gentle contact. The coated samples were then examined using a JSM-840 transmission electron microscope (JEOL).

## 3. Results and discussion

### 3.1. Optimization of propidium monoazide treatment conditions

VBNC cells are now recognized to coexist with culturable and dead cells in bacterial cultures grown in standard laboratory conditions [2,3,38]. To overcome the challenge of amplifying DNA from dead cells, a DNA modifier dye called PMAxx was employed [29]. Previous investigations have identified an optimal concentration range of 2–100 µM PMA for accurately determining the viability of various bacterial species in different sample matrices [5,29,39,40]. Establishing the appropriate concentration of PMAxx is crucial for precise quantification of viable cells and to avoid potential underestimation or false-negative outcomes. Use of an excessively high concentration of PMAxx can inhibit DNA amplification from viable cells, resulting in an underestimation of viable cell count. Conversely, a low concentration may not effectively suppress the signal from dead cells, leading to overestimation. Hence, it is important to determine the optimal concentration of PMAxx to ensure reliable assay performance.

**Figure 2.**
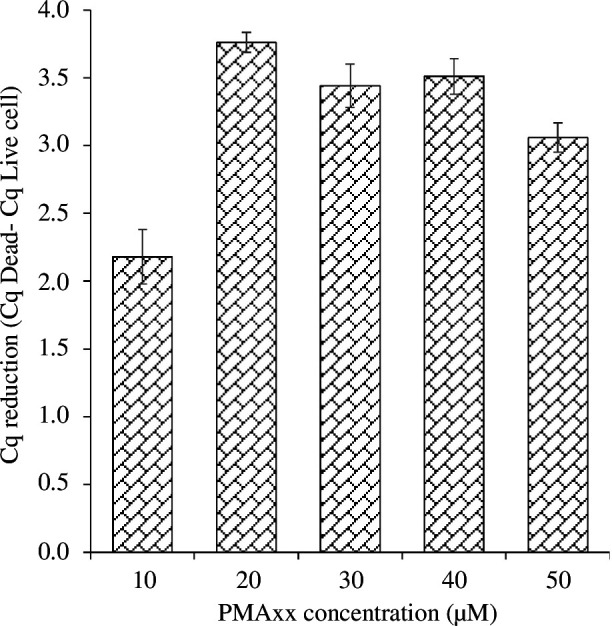
Optimization of the PMAxx concentration for detection of *C. concisus* ATCC 51562. Cq reductions were obtained by subtracting the mean Cq value obtained from PMAxx-treated dead cell aliquots from the mean Cq values from non-PMAxx-treated dead cells. Error bars in diagrams represent s.d. from three independent replicates.

To establish the optimal PMAxx concentration for inhibiting DNA amplification from dead *C. concisus* ATCC 51562 cells in qPCR, cells were treated with a range of PMAxx concentrations (0, 10, 20, 30, 40 and 50 µM) prior to DNA extraction. PMAxx-qPCR was then performed. The greatest reduction in Cq values was observed with a PMAxx concentration of 20 µM (figure 2). Therefore, for subsequent studies, a concentration of 20 µM PMAxx was selected as it effectively inhibited DNA amplification from dead *C. concisus* cells without significantly impacting the quantification of viable bacterial cells. Xiaonan *et al*. [5] have reported that 20 µM PMAxx was sufficient to inhibit signals from dead *C. jejuni* cells for the quantitative determination of cells in the VBNC state.

### 3.2. Standard curve for real-time quantitative PCR

**Figure 3.**
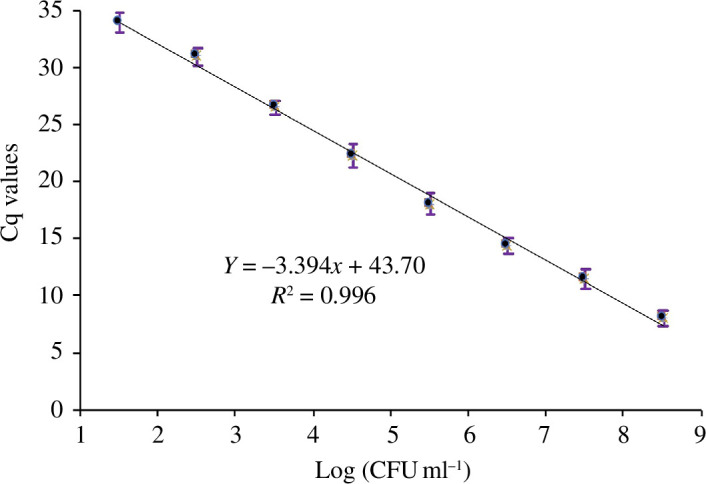
A standard curve of *C. concisus* ATCC 51562 concentration where mean Cq values were plotted against log_10_(CFU ml^−1^) of bacterial standard solution. The linear equation of the regression line and the coefficient of determination (*R* ^2^) are displayed in the graph.

To determine the efficiency of the qPCR, a standard curve was generated by performing serial dilutions of genomic DNA from *C. concisus* ATCC 51562, covering a range of 10^8^–10^2^ CFU ml^−1^. As shown in figure 3, a negative correlation was observed between the number of bacterial cells (log_10_ CFU ml^−1^) and the Cq value obtained from qPCR analysis of culturable *C. concisus* cells. The standard curve demonstrated linearity within the range of 1.5–8.5 log CFU ml^−1^ with an *R* ^2^ value of 0.996 (figure 3). The efficiency of qPCR amplification (*E*) was calculated to be 99.6%.

### 3.3. Validation of the optimized PMAxx-qPCR assay

**Figure 4.**
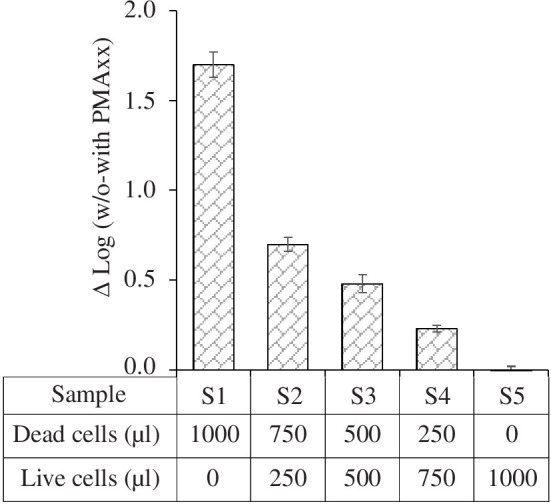
Suppression of DNA amplification from dead cells after PMAxx treatment in live/dead cell mixtures. The table presents the ratios at which viable and dead cells were combined. Results are shown as differences in ΔLog(without–with PMA) values by subtracting the log(CFU ml^−1^) values of PMAxx-treated from the log(CFU ml^−1^) values of PMAxx-untreated samples. The error bars represent the s.d. derived from three independent replicates. S represents sample no.

To assess the capability of the PMAxx method to distinguish between viable and heat-inactivated *C. concisus* cells, a set of mixtures containing different proportions of live and dead cells, as depicted in figure 4, were subjected to treatment with a concentration of 20 µM PMAxx. The number of gene copies obtained from PMAxx-treated cells were subtracted from those of non-treated cells. The number of gene copies without PMA showed no differences within a mixture of viable and dead cells from *C. concisus* (*t*‐test, *p* > 0.05), whereas the number of gene copies with PMAxx increased gradually with increasing proportions of viable cells resulting in ΔLog_10_ values decreasing (figure 4). These findings demonstrate the successful quantification of viable cells in mixtures containing both live and heat-killed *C. concisus* cells using PMAxx-qPCR.

### 3.4. Induction of the viable but nonculturable state under starvation and low temperature

**Figure 5.**
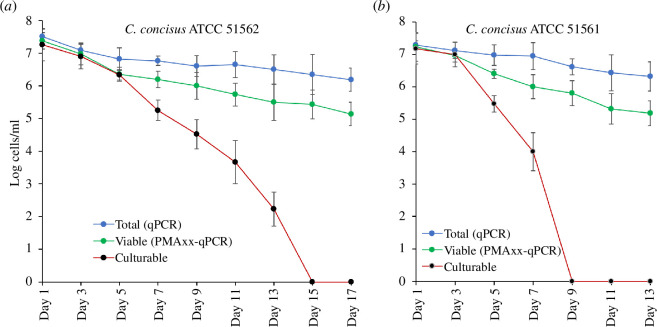
Entry into the VBNC state of *C. concisus* ATCC 51562 (*a*) and ATCC 51561 (*b*) under nutrient starvation conditions at low temperature (4°C). Blue circles (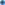) represent the total cell count (Group T) quantified by real-time qPCR. Green circles (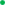) represent the viable cell counts (Group V) quantified using PMAxx-qPCR, while black circles (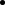) represent culturable cells (Group C) counted by culture-based methods. The error bars were calculated based upon three replicates.

*C. concisus* showed a variable timeline to enter into the VBNC state (figure 5). *C. concisus* ATCC 51562, a member of GS1, entered into the VBNC state in 15 days, while another strain, ATCC 51561, a GS2 strain, entered into the VBNC state in 9 days when incubated at 4°C in limited nutrient conditions. During the entire duration, the total cell counts consistently remained close to the initial level of 10^7^ CFU ml^−1^, indicating that total viability remained almost constant while standard traditional viability dropped. The VBNC state has been observed in more than 100 bacterial species when exposed to unfavourable environmental conditions such as heat, oxidative stress, antibiotics and other stressors [3]. Within the *Campylobacter* genus, it has been documented that four species, namely *C. jejuni*, *C. hepaticus*, *C. coli* and *C. lari*, can enter the VBNC state [3,7,8]. *C. jejuni* cells become VBNC in 10 days when incubated in low nutrient conditions (PBS) at 4°C [5]. However, under osmotic stress, the bacterium quickly enters the VBNC state within 48 h [5]. Another study reported that approximately 80% of the total population of *C. jejuni*, *C. coli* and *C. lari* entered into the VBNC state within 3 days when kept at simulated aquatic conditions at 10°C [41]. *C. hepaticus* enters into the VBNC state in 55 days when incubated in an isotonic solution at low temperature (4°C) [14].

Previous studies also reported that the survivability of *C. concisus* is temperature dependent [42,43] and that GS2 strains are better adapted to the human gut environment [44]. *C. concisus* survived for up to 6 days in saliva samples at low temperatures (4°C) [42]. In another study, bacterial numbers were not affected when clinical samples were tested after 7 h storage at 4°C [43]. Consequently, there is speculation that *C. concisus*-contaminated food or beverages, especially those subjected to refrigeration, may serve as a plausible source of infection [42]. There are some limitations in the study. A limited number of strains were investigated at a temperature of only 4°C under starvation. Effects of other factors such as osmotic stress, chemical stress, low pH, high temperature or a combination of multiple factors to induce VBNC in *C. concisus* need to be investigated.

### 3.5. Resuscitation of viable but nonculturable cells and analysis of morphological changes

A distinct and significant trait displayed by the VBNC cells is their ability to undergo *in vitro* resuscitation, wherein they can regain their cell division capability along with heightened metabolic activity, pathogenic potential and cellular morphology changes [14]. The resuscitation mechanisms vary across bacterial species. Besides the removal of stress, some bacterial species require additional triggers such as physical, chemical or host-related stimuli to recover from the VBNC state to a cultivable condition [13,14]. The well-characterized Gram-negative bacterium *Escherichia coli* was resuscitated from the VBNC state after temperature change [14]. Several studies have reported the resuscitation of *C. jejuni* and *C. coli* VBNC cells using experimental animal models [13]. Another member of *Campylobacter* species, *C. hepaticus*, was successfully resuscitated from VBNC to a culturable state using base media supplemented with a mixture of Vitox, FBP (ferrous sulfate, sodium metabisulfite and sodium pyruvate) and ʟ-cysteine [8]. In this study, *C. concisus* VBNC cells were resuscitated by subjecting them to a temperature upshift to 37°C, pre-enrichment and culturing in modified CA media containing sodium pyruvate, sodium metabisulfite and ferrous sulfate. Pyruvate is known to be one main factor that promotes the resuscitation of VBNC cells as they act as reactive oxygen species scavengers [14]. Pyruvate plays an important role in protecting *C. jejuni* from high oxygen stress and facilitates its growth [45]. Recently, it has been reported that pyruvate stimulates DNA and protein biosynthesis in *E. coli* VBNC cells and eventually supports growth restoration and colony formation [46]. Detailed studies including genomic and proteomic analysis are warranted to explore the underlying mechanisms in *C. concisus* VBNC cell resuscitation.

**Figure 6.**
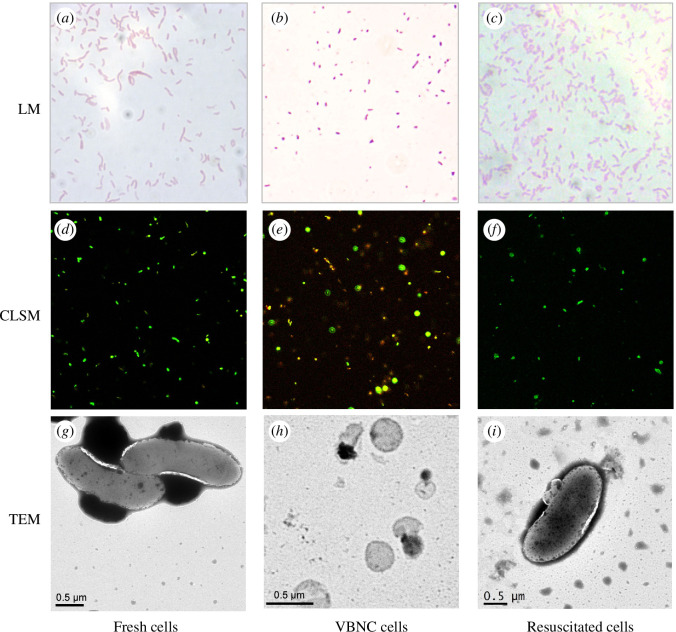
Morphological characteristics of *C. concisus* ATCC 51561 were examined under a light microscope (LM, 1000× magnification), a confocal laser scanning microscope (CLSM, 200× magnification) and a transmission electron microscope (TEM). Cells were stained with SYTO-9 and PI for CLSM analysis. LM (*a*–*c*), CLSM (*d*–*f*), TEM (*g*–*i*). Exponential-phase cells (*a*,*d*,*g*), VBNC cells (*b*,*e*,*h*), and resuscitated cells (*c*,*f*,*i*). VBNC cells (*h*) were round in shape and resuscitated cells (*i*) were returned to the regular shape of *C. concisus*.

The morphology of *C. concisus* resuscitated cells was examined and compared to VBNC and to normal, freshly grown, live cells. Significant morphological alterations were observed between cells in the exponential growth phase and those in the VBNC state. During the exponential phase, the cells displayed a characteristic rod or arc shape, with elongated forms clearly discernible under light microscope (1000×) and epifluorescence microscope (figure 6*a*,*d*). In contrast, in the VBNC state, a majority of the cells underwent a transformation into short rods or cocci, exhibiting irregular and distorted cell morphologies (figure 6*b*,*e*). Similar metamorphic changes were corroborated by transmission electron microscopy (figure 6*g*,*h*). Under TEM, it was observed that cells in the exponential phase had an average size of 4 × 0.5 μm (length × width), whereas the VBNC cells were smaller, measuring approximately 0.6 × 0.4 μm. Furthermore, LM, CLSM and TEM were employed to observe the morphological characteristics of resuscitated cells (figure 6*c,f*,i). The morphology of resuscitated cells was similar to *C. concisus* cells in exponential growth but larger than the VBNC cells.

In previous studies, when *C. jejuni* entered the VBNC state, curved-shaped cells were converted to coccoid-shaped cells [47]. Morphological transformation, from comma-shaped cells to coccoid-shaped cells, has also been noted in *H. pylori* upon entry into the VBNC state [31]. VBNC cells possess a remarkable ability to undergo resuscitation both in controlled laboratory conditions [9] and in natural estuarine environments, triggered by environmental alterations such as temperature elevation. Notably, *Vibrio alginolyticus* was resuscitated from the VBNC state within 16 h by subjecting to a temperature upshift and natural estuarine environments owing to increased temperature [3,14,32]. In another study, researchers were able to retrieve the causative agent of spotty liver disease in chickens, *C. hepaticus*, from its VBNC state using an enrichment medium [8]. The findings of the current study highlight the importance of pre-enrichment in a nutrient-rich broth for the successful resuscitation of *C. concisus*.

## 4. Conclusion

The findings demonstrate that *C. concisus* has the ability to enter a VBNC state under stressful environmental conditions. The ATCC51561 strain belonging to GS2, which is known to be better adapted to gut environment, entered the VBNC state earlier than ATCC51562, a GS1 strain. The VBNC cells can be resuscitated through a temperature upshift in the presence of nutrients. Upon resuscitation, they exhibit a morphology similar to that of normal cells. Optimal conditions were established for the application of PMAxx to effectively inhibit signals from dead cells during PCR amplification. The use of PMAxx-qPCR provides a reliable method for the specific detection and quantification of viable and VBNC cells of *C. concisus*. This method can, in the future, be applied to accurately determine the viable bacterial load in clinical and environmental samples. Taken together, the findings suggest that the VBNC state could play a significant role in the survival and transmission of *C. concisus* in the oral cavity and the GI tract.

## Data Availability

Raw data generated from this study are included in the four files relevant to figures 2, 3, 4 and 5 which can be accessed through: https://doi.org/10.5061/dryad.0cfxpnw8v [48].
